# Understanding the diagnosis of catheter-related bloodstream infection: real-time monitoring of biofilm growth dynamics using time-lapse optical microscopy

**DOI:** 10.3389/fcimb.2023.1286527

**Published:** 2023-12-06

**Authors:** Marta Díaz-Navarro, Rafael Samaniego, Juan Carlos Piqueras, Rafael Díez, Rama Hafian, Irene Manzano, Patricia Muñoz, María Guembe

**Affiliations:** ^1^ Department of Clinical Microbiology and Infectious Diseases, Hospital General Universitario Gregorio Marañón, Madrid, Spain; ^2^ Instituto de Investigación Sanitaria Gregorio Marañón, Madrid, Spain; ^3^ Confocal Microscopy Unit, Instituto de Investigación Sanitaria Gregorio Marañón, Madrid, Spain; ^4^ School of Biology, Universidad Complutense de Madrid, Madrid, Spain; ^5^ CIBER Enfermedades Respiratorias-CIBERES (CB06/06/0058), Madrid, Spain; ^6^ School of Medicine, Universidad Complutense de Madrid, Madrid, Spain

**Keywords:** catheter-related bloodstream infections, biofilm, differential time to positivity, time-lapse optical microscopy, growth

## Abstract

**Background:**

The differential time to positivity (DTTP) technique is recommended for the conservative diagnosis of catheter-related bloodstream infection (C-RBSI). The technique is based on a 120-minute difference between microbial growth in blood drawn through the catheter and blood drawn through a peripheral vein. However, this cut-off has failed to confirm C-RBSI caused by *Candida* spp. and *Staphylococcus aureus*.

**Objective:**

We hypothesized that the biofilm of both microorganisms disperses faster than that of other microorganisms and that microbial load is rapidly equalized between catheter and peripheral blood. Therefore, our aim was to compare the biofilm dynamics of various microorganisms.

**Methods:**

Biofilm of ATCC strains of methicillin-resistant *Staphylococcus epidermidis*, methicillin-susceptible *S. aureus*, *Enterococcus faecalis*, *Escherichia coli* and *Candida albicans* was grown on silicon disks and analyzed using time-lapse optical microscopy. The time-lapse images of biofilms were processed using ImageJ2 software. Cell dispersal time and biofilm thickness were calculated.

**Results:**

The mean (standard deviation) dispersal time in *C. albicans* and *S. aureus* biofilms was at least nearly 3 hours lower than in biofilm of *S. epidermidis*, and at least 15 minutes than in *E. faecalis* and *E. coli* biofilms.

**Conclusion:**

Our findings could explain why early dissemination of cells in *C. albicans* and *S. aureus* prevents us from confirming or ruling out the catheter as the source of the bloodstream infection using the cut-off of 120 minutes in the DTTP technique. In addition, DTTP may not be sufficiently reliable for *E. coli* since their dispersion time is less than the cut-off of 120 minutes.

## Introduction

Bacterial and fungal biofilms are responsible for chronic infections associated with biomedical devices, which represent a serious public healthcare problem (Veerachamy et al., 2014; Tascini et al., 2018). Biofilm infections are caused by adhesion of bacteria or fungi to the surfaces of devices, such as catheters. Biofilm are composed of an extracellular polymeric matrix with very selective permeability to antimicrobial agents (Høiby et al., 2015; Bjarnsholt et al., 2018; Wolcott, 2021). Thus, the nature of biofilm structure and the physiological characteristics of cells embedded in biofilm confer high resistance to antibiotic agents and the immune response, making it difficult to eradicate the infection. Furthermore, the superficial layers of the biofilm are formed by sessile cells, which may detach and disperse toward other tissues (Ferretti et al., 2002; Ferrer and Almirante, 2014; Gahlot et al., 2014; Gominet et al., 2017; Lopes et al., 2019). These results in the appearance of persistent cells and the detachment of individual cells or microcolonies from biofilm, which can hamper diagnosis and treatment (Høiby et al., 2015; Di Domenico and Oliva, 2022).

When sessile cells from the upper layer of the biofilm formed on the catheter surface disperse into the bloodstream, they cause catheter-related bloodstream infection (C-RBSI), one of the most important nosocomial infections (Donlan, 2002; Maki et al., 2006; Chaves et al., 2018; Cangui-Panchi et al., 2022). When suspected C-RBSI must be confirmed before catheter removal, the recommended conservative diagnostic technique is differential time to positivity (DTTP) (Mermel et al., 2009; Chaves et al., 2018). This technique consists of simultaneously obtaining blood cultures from all catheter lumens and from a peripheral vein to determine microbial growth using an automated system. As microbial load from blood obtained through the catheter is higher when the catheter is the source of the infection, growth could be detected more quickly than in blood obtained from the bloodstream. A difference in the time to positivity of 120 minutes between blood obtained from the catheter and blood obtained from the peripheral vein has been established as a cut-off to microbiologically confirm C-RBSI (Sabatier et al., 2015; Chaves et al., 2018; Ruiz-Giardin et al., 2019; Wolcott, 2021). However, discrepancies in this technique have been reported when C-RBSI is caused by specific microorganisms, such as *Candida* spp. and *Staphylococcus aureus* (Park et al., 2010; Bouza et al., 2013; Fernández-Cruz et al., 2014; Kaasch et al., 2014; Bouzidi et al., 2018; Gits-Muselli et al., 2020; Orihuela-Martín et al., 2020).

The hypothesis of our study was that dispersal of cells from the biofilm differs depending on the microorganism and may be faster in *Candida* spp. and *S. aureus* in compare with other relevant microorganisms frequently caused of C-RBSI, such as other gram positive microorganisms. As a result, concentrations of yeasts/bacteria are equalized in both the catheter and the blood compartment in early stages of infection, thus preventing the DTTP cut-off of 120 minutes from being applied in these species.

Our objective was to establish the cell spreading time and, complementarily, the thickness of the biofilm from *Candida albicans*, *S. aureus*, *S. epidermidis*, *E. faecalis* and *E. coli* using time-lapse optical microscopy, which enables real-time visualization of biofilm and living species.

## Materials and methods

The study was carried out in the Microbiology Laboratory of Hospital General Universitario Gregorio Marañón, Madrid (Spain).

### Strains and culture conditions

We selected 5 American Type Culture Collection (ATCC) strains of microorganisms frequently isolated from C-RBSI (Rodríguez-Créixems et al., 2013): methicillin-resistant *Staphylococcus epidermidis* (MRSE) ATCC35984 (MRSE), methicillin-susceptible *Staphylococcus aureus* (MSSA) ATCC29213 (MSSA), *Enterococcus faecalis* ATCC35186, *Escherichia coli* ATCC 25922 and *Candida albicans* ATCC14053 (as the most representative specie of *Candida* spp. causing C-RSBI in our hospital) (Pérez-Parra et al., 2009; Guembe et al., 2010). All of the strains were stored at –80°C until use.

First, an inoculum of each strain was obtained after culture of 24 hours at 37°C in the corresponding enrichment medium (Tryptic Soy Broth [TSB] supplemented with 1% glucose for *S. epidermidis* and *E. faecalis*, TSB for *S. aureus*, Yeast Peptone Dextrose [YPD, composed of yeast extract and peptone, and D-glucose monohydrate in sterile water] and Roswell Park Memorial Institute [RPMI-1640, with L-glutamine and morpholinepropanesulfonic acid, MOPS] for *C. albicans*, and Luria-Bertani Broth [LB] for *E. coli*). All mediums were purchased from Thermo Fisher Scientific and Sigma-Aldrich. The samples were processed in an orbital shaker at 150 rpm and 37°C for 24 hours. Then, 3 centrifugation washes were performed with phosphate-buffered saline (PBS; 0.1M, pH 7.4). A cell suspension was inoculated into the corresponding medium, which was adjusted to a concentration of 10^8^ CFU/ml using a 0.5 McFarland scale (10^6^ CFU/ml of 0.35 McFarland scale for *C. albicans*).

### Biofilm growth on silicon disks

**Figure 1:**
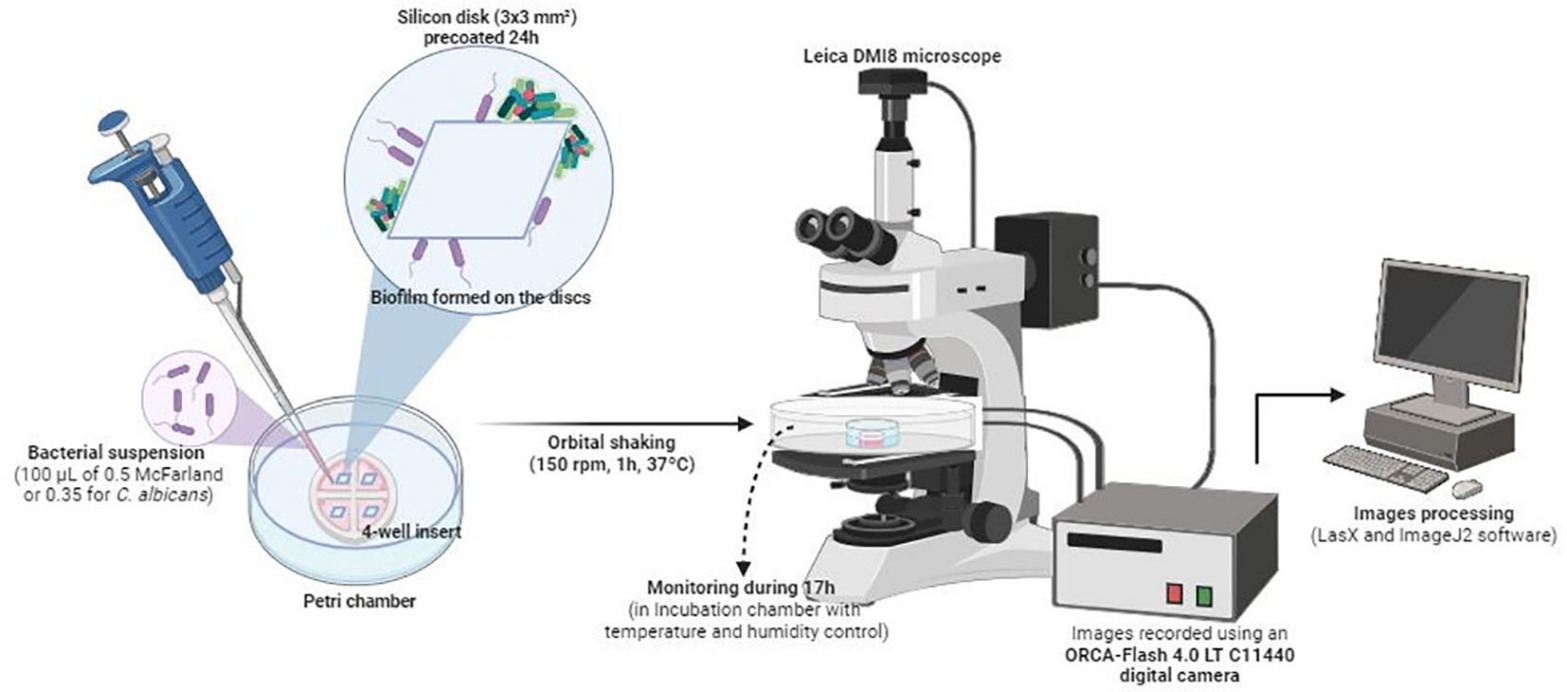
Schematic representation of the *in vitro* biofilm formation procedure and time-lapse images analysis.

Biofilms were grown under aerobic conditions on silicon disks measuring 3 × 3 mm^2^ in area and 0.38 mm in thickness (Merck Life Science, S.L.) according to what it previously reported (Kaneko et al., 2013). These were pretreated for 24 hours at 37°C with poly-L-lysine to promote initial adhesion of bacteria or yeast. Then, for time-lapse imaging, disks were washed with PBS and individually placed in 35-mm glass-bottom Petri chambers, each mounted with a 4-well insert (**Figure 1**). The total area and volume available in each well were 0.35 cm^2^ and 110 µl (Ibidi GmbH, Martinsried, Germany).

Next, 100 µl of each cell suspension was added to the wells and incubated for 1 hour at 150 rpm and 37°C to prevent precipitation of the bacteria or yeast to the bottom of the chamber by gravity.

### Time-lapse optical microscopy analysis

Petri chambers were maintained in an incubation chamber for *in vitro* experiments. Both temperature (37°C) and humidity were monitored.

Biofilm formation was recorded using an ORCA-Flash 4.0 LT C11440 digital camera mounted on a Leica DMI8 microscope (Leica Geosystems AG, Heerbrugg, Switzerland) with an ACS-APO 63x glycerol immersion objective (**Figure 1**). Time-lapse images of biofilm were recorded at a rate of 1 frame per minute for *S. aureus*, *S. epidermidis*, *E. faecalis*, and *E. coli* and per 1.5 minutes for *C. albicans*, beginning after 1 hour of shaking at 150 rpm to promote the initial attachment. Biofilm development was monitored for 17 hours for three technical replicates for each microorganism. Images were processed using ImageJ2 software (Rueden et al., 2017). Biofilm thickness was measured hourly after 1 hour of incubation and up to 17 hours of development from three biofilms grown on three silicon discs. In addition, the three biofilms have already been monitored to get sessile cell dispersal times of each microorganism, corresponding to the first three events in which we observed cells’ detachment within the biofilm aggregates. Data were plotted using GraphPad Prism® version 8.0 for Windows, GraphPad Software, www.graphpad.com. As we aimed to observe differences between a young and a mature attached biofilm, we compared a short (1 hour) vs. a long (17 hours) timepoint.

### Definitions

We considered a mature biofilm when we observed a dense (dark) network of hyphae (in *C. albicans*) or cells (in bacteria). This fact indicates the presence of a good extracellular matrix (Kaneko et al., 2013).

we considered a young biofilm when single cells formed few and small aggregates, without the presence of such as a dense mass.

### Statistical analysis

We compared the variables analyzed, dispersal time and biofilm thickness, from both *C. albicans* and *S. aureus* (test species) with those from *S. epidermidis*, *E. faecalis*, and *E. coli* (control species), and also between control species themselves.

The non-parametric Kruskal-Wallis test and Mann-Whitney test was applied to analyze statistical differences between groups. Significance values were adjusted by Bonferroni correction. Statistical significance was set at *p*<0.05. Results are expressed as the mean ± standard deviation (SD) of three measurements taken from three different biofilms grown on silicon discs.

## Results

### Biofilm formation

#### Candida albicans

**Figure 2:**
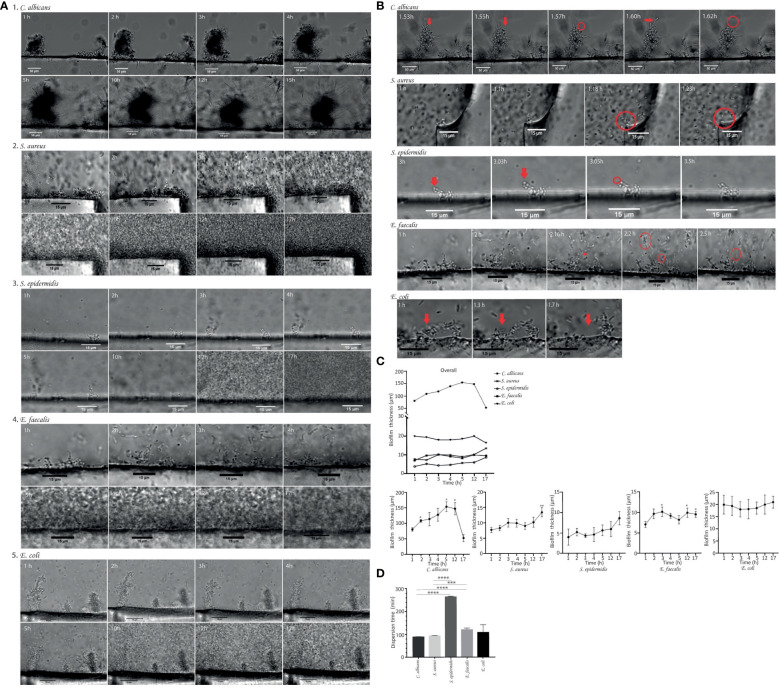
**(A)** Time-lapse images (63× magnification) showing the development of biofilms of *Candida albicans* ATCC14053, *Staphylococcus aureus* ATCC29213, *Staphylococcus epidermidis* ATCC35984, *Enterococcus faecalis* ATCC35186 and *Escherichia coli* ATCC25213. Growth dynamics of biofilm on silicon disks after 1 hour of incubation under orbital shaking shown as time-lapse images taken hourly during the 17-hour follow-up. **(B)** The moment of detachment of cell clusters and/or single cells (red arrows) from the biofilms indicating the dispersal phenomenon. The scale bar represents 50 µm to (*C*) *albicans* and 15 µm to *S. aureus*, *S. epidermidis*, (*E*) *faecalis* and (*E*) *coli*. **(C)** Graphical representation of the growth dynamics of *Candida albicans* biofilm, *Staphylococcus aureus* biofilm, *Staphylococcus epidermidis* biofilm, *Enterococcus faecalis* biofilm and *Escherichia coli* biofilm. Data shown biofilms on three different areas of silicon disks by hourly quantification of biofilm thickness in µm over 17 hours. Mean ± SEM is shown. The y-axis shows biofilm thickness in µm. The x-axis shows the monitoring time in hours. Significant differences respective to the first time point (*, *p*<0.05, Student´s *t* test). **(D)** Biofilm dispersal time. The graph shows the comparison of biofilm dispersal times for each strain studied in minutes. These are indicated in the figure legend and include *Candida albicans*, *Staphylococcus aureus*, *Staphylococcus epidermidis*, *Enterococcus faecalis* and *Escherichia coli*. The longest dispersal time was recorded for *S. epidermidis*, followed by *E. faecalis* (above 120 min in both cases). Data are shown as the mean ± SEM from three different areas of three silicon disk.

**Figure 2A** shows sequential images (at 63× magnification) of *C. albicans* biofilm growth taken at 60-seconds intervals using time-lapse optical microscopy. The time lapse images revealed the presence of yeast that had already adhered to the silicon disk after 1 hour of incubation, with mature biofilm observed on the surface. Within 17 hours of follow-up, *C. albicans* biofilm gradually increased in mean (± SD) thickness from 80.68 ± 9.49 µm at 1 hour to 147.50 ± 19.51 µm at 12 hours (**Table 1**; **Figure 2A, C**). After 12 hours, the average biofilm thickness decreased, probably because of massive detachment of cell clusters (**Table 1**; **Figure 2C**). Time-lapse optical microscopy images revealed that *C. albicans* biofilm developed in a stepwise fashion and that it was a dynamic process, since attachments and detachments occurred simultaneously.

**Table 1:** Comparison of biofilm thickness (µm, mean ± SD) and dispersal time (min, mean ± SD) by microorganism.

Microorganism	Mean^a^ ± SD thickness (µm) at 1 h incubation				Mean^a^ ± SD thickness (µm) at 17 h incubation				Mean^a^ ± SD dispersal time (min)			
	*C. albicans*	*P*	*S. aureus*	*p*	*C. albicans*	*P*	*S. aureus*	*p*	*C. albicans*	*p*	*S. aureus*	*p*
*S. epidermidis*	80.68 ± 9.49 4.00 ± 3.43	0.050	7.75 ± 2.28 4.00 ± 3.43	0.275	52.61 ± 22.18 8.61 ± 5.55	0.047	12.97 ± 2.77 8.61 ± 3.55	1.000	90.17 ± 0.44 265.70 ± 1.01	0.050	95.23 ± 0.43 265.70 ± 1.01	0.046
*E. faecalis*	80.68 ± 9.49 7.06 ± 2.37	0.050	7.75 ± 2.28 7.06 ± 2.37	0.827	52.61 ± 22.28 9.91 ± 2.52	0.047	12.97 ± 2.77 9.91 ± 2.52	1.000	90.17 ± 0.44 122.30 ± 2.48	0.050	95.23 ± 0.43 122.30 ± 2.48	0.046
*E. coli*	80.68 ± 9.49 19.97 ± 8.66	0.050	7.75 ± 2.28 19.97 ± 8.66	0.127	52.61 ± 22.28 20.74 ± 12.77	1.000	12.97 ± 2.77 20.74 ± 12.77	1.000	90.17 ± 0.44 109.83 ± 18.74	0.513	95.23 ± 0.43 109.83 ± 18.74	0.507

**^a^** Mean of three different silicon disks; SD, standard deviation.

#### Staphylococcus aureus

In the case of *S. aureus*, the development of biofilm was also a dynamic process.

The growth pattern in *S. aureus* was similar to that of *C. albicans*, with a gradual increase in mean ( ± SD) thickness from 1 hour (7.75 ± 2.28 µm) up to 17 hours (12.97 ± 2.77 µm) (**Table 1**; **Figures 2A, C**). Moreover, the *S. aureus* biofilm reached a significant early biomass at 3.5 hours on all disk surfaces. The time-lapse images revealed that *S. aureus* biofilm was continually growing and shedding in some areas throughout the 17-hour monitoring period. It means that detachment does not occur equally throughout the biofilm adhered on surface. While in some areas the biofilm continues to increase in thickness, in other areas detachment of individual cells or aggregates occurred.

#### Staphylococcus epidermidis

In the case of *S. epidermidis* biofilm, time-lapse images revealed that cells take longer to attach to the surface (**Figures 2A, C**) and thus to form a mature biofilm. *S. epidermidis* biofilm consisted of small aggregates not uniformly distributed over the disk surface and showed lower thicknesses at all time-points over a 17-hour period: 4.00 ± 3.43 at 1 hour, 4.62 ± 1.73 µm at 4 hours, and 8.61 ± 5.55 µm at 17 hours (**Table 1**).

#### Enterococcus faecalis

The sequential images of *E. faecalis* growth show a non-continuous biofilm consisting in small aggregates with a mean thickness of 7.06 ± 2.37 µm adhered to the disk surface 1 hour after inoculation (**Table 1**; **Figures 2A, C**). *E. faecalis* developed progressively until 2.5 hours, with the mean thickness increasing to 10.16 ± 2.59 µm. After 2.5 hours, the thickness of the biofilm remained around 9 µm and unchanged up to 17 hours of follow-up (**Figures 2A, C**).

#### Escherichia coli

The time-lapse images at 1 hour after inoculation revealed the presence of *E. coli* biofilm, which consisted of macrocolonies with a mean (± SD) thickness of 19.97 ± 8.66 µm. We observed a stable biofilm thickness over 17 hours of monitoring, with a slight increase in mean thickness of 0.77 ± 7.12 µm (**Table 1**; **Figures 2A, C**). Although a well-formed biofilm was observed after 1 hour of inoculation, the surface of the silicon disk was not completely covered by biofilm until 5 hours later.

#### Comparisons

The mean thickness of *C. albicans* biofilm after 1 hour of inoculation was significantly higher than that of the 3 reference species (*S. epidermidis*, *E. faecalis*, and *E. coli*) (*p*=0.050) (**Table 1**). We found no statistically significant differences in terms of biofilm thickness at 1 hour between bacterial species (*p*>0.05) (**Table 1**).

Furthermore, biofilms of *C. albicans* and *S. aureus* developed more rapidly, forming mature biofilm in a shorter time, occupying the whole silicon disk surface (**Figure 2A**), whereas *S. epidermidis* and *E. faecalis* formed biofilms consisting of small aggregates not uniformly distributed on the disk. This differed from *E. coli*, which also formed thicker aggregates distributed uniformly on the disk surface (**Figure 2A**).

### Dispersal of biofilm cells

Both *C. albicans* and *S. aureus* biofilm cells dispersed by detachment in clusters from the superficial layer (**Figure 2B**). In the case of *C. albicans* biofilm, these aggregates detached from the hyphae tips. In both biofilms, the average mean ( ± SD) dispersal time was similar, that is, 90.00 ± 0.29 minutes for *C. albicans* and 95.23 ± 0.43 minutes for *S. aureus* (**Table 1**).

In *E. coli*, small aggregates detached after a mean ( ± SD) dispersal time of 109.83 ± 18.74 minutes, along with release of single cells in some areas (**Table 1**; **Figure 2B**).

In *E. faecalis*, biofilm cells also dispersed in clusters (**Figure 2B**), with a mean (± SD) dispersal time of 122.30 ± 2.48 minutes (**Table 1**).

By contrast, the dispersal pattern for *S. epidermidis* biofilm was mostly through single cells from the superficial layer into the surrounding layers and took longer (**Figure 2B**), with the mean (± SD) dispersal time being 265.70 ± 1.01 minutes (**Table 1**).

#### Comparisons

The dispersal time of *C. albicans* and *S. aureus* biofilm was significantly shorter than that of the reference microorganisms *S. epidermidis* and *E. faecalis* (p<0.001), although no statistically significant differences were detected between *E. coli* and *C. albicans* (p=0.353) or between *E. coli* and *S. aureus* (p=0.480) (**Table 1**; **Figure 2D**).

## Discussion

Our results revealed that, based on faster biofilm dispersal, the cut-off of 120 minutes for the DTTP technique may not be equally applicable to confirm or rule out the catheter as the source of bloodstream infection in all microorganisms tested (*C. albicans*, *S. aureus*, *S. epidermidis*, *E. faecalis* and *E. coli*). Specifically, the DTTP cut-off may not be discriminatory when C-RBSI is caused by *C. albicans*, *S. aureus*, or, presumably, *E. coli.*

According to data provided by prevalence studies of nosocomial infections, between 5% to 15% patients admitted to European and North American hospitals are affected by nosocomial infections, being C-RBSI one of the most frequent, which is associated with high morbidity, mortality and healthcare cost (Alexandrou et al., 2018; Cangui-Panchi et al., 2022; Tabah et al., 2023). Early diagnosis of C-RBSI is essential if we are to prevent microorganisms spreading from the primary site of infection and invading other tissues, such as blood, irrespective of the source of acquisition (extra or intraluminal route), as etiology is similar. However, despite its importance, early diagnosis remains an unmet need in clinical microbiology, and our understanding of biofilm formation dynamics is limited by the lack of real-time detection techniques (Ascenzioni et al., 2021).

DTTP is currently the recommended technique for diagnosis of C-RBSI before catheter removal (Mermel et al., 2001; Mermel et al., 2009; Bouza et al., 2013; Ruiz-Giardin et al., 2019; Wiggers and Daneman, 2020; Wolcott, 2021). However, the validity of the established cut-off of 120 minutes has shown in the systematic review for Dhaliwal et al. worse results in terms of sensitivity and specificity to confirm *Candida* spp. and *S. aureus* C-RBSI, respectively (Dhaliwal and Daneman, 2023). Other previous studies also revealed low ability of DTTP to discriminate between non-C-RSBI and C-RBSI caused by *S. aureus* (Bouzidi et al., 2018; Orihuela-Martín et al., 2020). This may be explained by factors related to the speed of microbial growth and the formation and dispersal of biofilm causing the microbial load present in the catheter and blood to equalize rapidly. In contrast, another hypothesis may also be that the time window for correct assessment of C-RBSI will be shorter for a strain with fast dispersion. This becomes even more important in the case of *C. albicans* and *S. aureus*, which actually are some of the most frequent microorganisms causing C-RSBI (European Centre for Disease Prevention and Control, 2023).

In addition to the growth of biofilm after initial adhesion (Kaneko et al., 2013; Chong et al., 2021; Choong et al., 2021), the mechanism of biofilm dispersal plays an important role in the pathogenicity and dissemination of bacterial and fungal biofilm-associated infections (Hall-Stoodley et al., 2004; Penesyan and Paulsen, 2021). In the present study, time-lapse images enabled simultaneous observation and monitoring of growth, attachment, dispersal, and motion of single cells and biofilm microcolonies. We found that the formation of biofilm by all the microorganisms studied was a dynamic process in which the attachment and detachment phases converge. Similarly, the micro- and macrocolonies formed contributed to the development and expansion of the biofilm and cell clusters, and single cells were actively released and migrated along the surface before attaching to new areas of the disk, thus contributing to the formation of new biofilms, as it was already known (Otto, 2013; Lister and Horswill, 2014; Wall et al., 2019). Dispersal of *C. albicans* and *S. aureus* was mostly through the formation of clusters of yeast and bacteria, respectively. These clusters were actively released as clumps when they reached a considerable thickness, as previously reported (Hall-Stoodley et al., 2004). Another hypothesis, among others, are the formation of a concentration gradient along the full biofilm thickness or hypoxia conditions occurring in the deeper layers, which can lead to dispersal (Rumbaugh and Sauer, 2020). This type of dispersal via clusters may be more successful than dispersal via the loss of single cells within the biofilm, which occurred for *S. epidermidis*. Our results are consistent with those reported in previous studies, in which it was demonstrated that *S. epidermidis* biofilm is continually seeding the environment with individual bacterial cells in their planktonic form (Stoodley et al., 2001; Purevdorj-Gage et al., 2005; Wolcott, 2021). In contrast, a combination of both dispersal strategies, that is, small aggregates and single cell dispersal, occurred for *E. coli*.

To our knowledge, this is the first study to describe the real-time monitoring of biofilm dispersal according to species. Cell dispersal of *C. albicans* and *S. aureus* occurred faster than that of *S. epidermidis* and *E. faecalis*, with the mean (± SD) dispersal time in both species around 90 minutes (90.17 ± 0.44 min and 95.23 ± 0.43 min, respectively) versus 265.70 ± 1.01 minutes for *S. epidermidis* (*p*=0.050 and *p*=0.046) and 122.30 ± 2.48 minutes for *E. faecalis* (*p*=0.05 and *p*=0.050). Therefore, as cells disseminate early to the bloodstream, the yeast/bacteria concentrations are rapidly equalized both in catheter and peripheral blood, with the result that it is not possible to apply the DTTP cut-off of 120 minutes between both compartments.

We observed a mean dispersal time for *C. albicans* and *S. aureus* biofilm lower than the DTTP cut-off of 120 minutes, which was also similar in *E. coli* (109.83 ± 18.74 min). Furthermore, we observed a significantly lower mean ( ± SD) dispersal time for *E. coli* than for *S. epidermidis* (*p*=0.050). Flagella are essential for the adhesion of *E. coli* (Pratt and Kolter, 1998; Reisner et al., 2003) and formation of biofilm and may play a key role in its rapid dispersal. Although the incidence of Gram-negative bacilli C-RBSI is low (Guembe et al., 2017; Calò et al., 2020), it has been increasing during recent years (Fares et al., 2019; Ruiz-Ruigómez et al., 2020; Wiggers and Daneman, 2020). Based on a previous analysis of 145 cases of *E. coli* bacteremia with a suspicion of C-RBSI in our institution, DTTP only confirmed 14.5% as *E. coli* C-RBSI, with the remaining 85.5% being unrelated. However, we remain unsure of whether they were really not C-RBSI–related or whether the DTTP technique was unable to detect them, based on our hypothesis that *E. coli* biofilm grew so quickly that the cut-off of 120 minutes could not be applied (data not published). Therefore, given that we eventually removed only 5 catheters, future studies should attempt to corroborate our hypothesis based on data for withdrawn catheter tip cultures to confirm or rule out C-RBSI episodes.

In addition, we observed differences in biofilm structure between microorganisms. Given that yeasts are larger than bacteria, we expected to find differences in biofilm thickness between yeasts and bacteria. Therefore, *C. albicans* formed a significantly thicker biofilm than bacteria both at 1 hour and 17 hours of incubation. Biofilm thickness at 1 hour was significantly higher for *C. albicans* than for *S. aureus*, *S. epidermidis*, *E. faecalis*, and *E. coli* (*p*=0.050). We found no statistically significant differences between bacterial species at either 1 hour or 17 hours of incubation. In addition, this thickness may be closely related to dispersion since the thicker biofilm, the higher shear stress associated with the surface layers of the catheter biofilms and, therefore, the higher detachment events.

Furthermore, continuing with the structure of biofilms, while *S. aureus* and *C. albicans* formed an entire biofilm occupying the whole disk surface, *S. epidermidis* formed a biofilm that was irregularly distributed across the disk surface. The same occurred with respect to the biofilm structure of *E. faecalis* and *E. coli*, which was formed by microcolonies dispersed across the surface.

One of our limitations is that, as we only tested ATCC strains of the most common bacteria and fungi causing C-RBSI, further studies are needed to validate our findings using a wide range of clinical strains from affected patients, including clinical strains of non-*albicans Candida* spp., which have become increasingly important in recent years. Regarding C. albicans, as we observed aggregates at 1 h, and blastopores would not have enough time for duplicating, sonication could have help to put a homogenous yeast suspension, since a yeast clump will be able to disperse faster than individual yeasts. Furthermore, another limitation than can be noted is that we only measured biofilm thickness as the main parameter to contrast the difference of how biofilm is different between species. However, future studies are needed to assess additional parameters related to biofilm structure. In addition, we did not take account of other factors that are present in daily clinical practice, such as the presence of proteins, the immune response and the shear stress to which these catheter biofilms would be subjected, which may not mimic the clinical scenario of catheter-related infections. And also, the heterogeneity of the biofilm-inducing media used could be a problem. However, we used the optimal biofilm-inducing growth media for each microorganism, as we consider that rich growth media may be relevant for biofilm formation. We consider it is needed to use different growth media for each specie in particular to create the best conditions and nutrients. If we would have used the same media for all microorganisms, they would have shown different growth kinetics, and therefore, this would have mean a potential bias in the study (Woitschach et al., 2022).

## Conclusions

Our is the first *in vitro* study to assess differences in the time to biofilm dispersal between microorganisms frequently causing C-RBSI. The rapid dispersal of *C. albicans* and *S. aureus* compared with *S. epidermidis* and *E. faecalis* may explain the lack of application of the DTTP technique in the diagnosis of C-RBSI. Moreover, as *E. coli* also rapidly disperses within the biofilm, future studies are needed to assess the reliability of DTTP in *E. coli* C-RBSI.

## Data availability statement

The original contributions presented in the study are included in the article/**Supplementary Material**. Further inquiries can be directed to the corresponding author.

## Author contributions

MD: Conceptualization, Formal Analysis, Methodology, Software, Writing – original draft. RS: Formal Analysis, Software, Writing – original draft. JP: Methodology, Writing – original draft. RD: Methodology, Writing – original draft. RH: Methodology, Writing – original draft. IM: Methodology, Writing – original draft. PM: Supervision, Validation, Writing – original draft. MG: Conceptualization, Funding acquisition, Resources, Supervision, Validation, Writing – review & editing.
