# Supplementary figures and images for: Understanding the diagnosis of catheter-related bloodstream infection: real-time monitoring of biofilm growth dynamics using time-lapse optical microscopy
Source: Front Cell Infect Microbiol. 2023 Dec 6;13:1286527. doi: 10.3389/fcimb.2023.1286527 (PMC10731284; doi:10.3389/fcimb.2023.1286527)

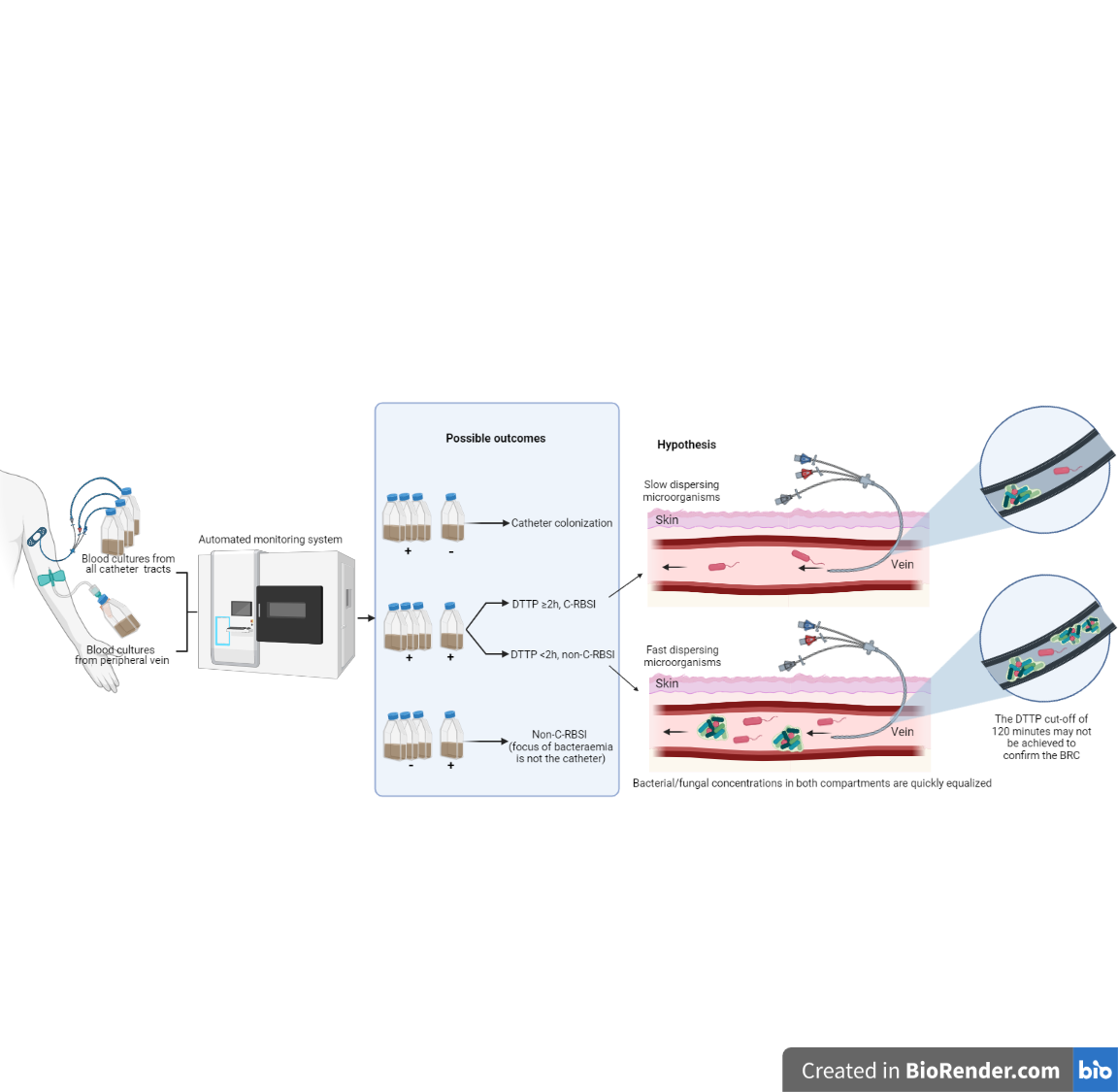

Supplement: Supplementary file 6 [file Image_1.png]
